# Adrenomedullin regulated by miRNA-574-3p protects premature infants with bronchopulmonary dysplasia

**DOI:** 10.1042/BSR20191879

**Published:** 2020-05-27

**Authors:** Xiaohui Gong, Jiajun Qiu, Gang Qiu, Cheng Cai

**Affiliations:** 1 Department of Neonatology, Shanghai Children’s Hospital, Shanghai Jiao Tong University, Shanghai 200062, People’s Republic of China; 2 Department of Informatics, I12-Chair of Bioinformatics and Computational Biology, Technical University of Munich (TUM), Boltzmannstrasse 3, Garching, Munich 85748, Germany

**Keywords:** BPD, preterm neonates, micoRNA-574-3P, adrenomedullin, protective

## Abstract

Bronchopulmonary dysplasia (BPD) is the most common chronic lung disease (CLD) in premature infants. The present study was designed to elucidate the regulation of miRNA-547-3p on adrenomedullin (ADM) during the pathogenesis of BPD. We used Agilent Human 4x44K Gene Expression Microarrays v2 and miRCURY LNA™ microRNA Array to identify the differently expressed miRNA and its potential target genes, and certified them again by luciferase reporter gene analysis. We only retained target genes that met the following two conditions: first, coexisting in two databases, and second, expressing differences, and then identifying target genes by luciferase reporter gene analysis. Thus, we selected miRNA-574-3p and its target gene ADM for further research. We used real-time q-PCR to determine the expression of miRNA-574-3p and its target gene ADM in premature infants with BPD. We used microarray expression to analyze BPD samples and non-BPD samples and found that there were 516 differently expressed probes between them. The 516 differently expressed probes included 408 up-regulated probes and 108 down-regulated probes. The blood samples of BPD infants were detected by real-time q-PCR and found that the expression of miRNA-574-3p was decreased, while the expression of ADM was significantly increased. Luciferase reporter gene analysis showed that hsa-miR-574-3p can regulate the expression of luciferase with ADM 3′UTR, and decrease it by 61.84%. It has been reported in the literature that ADM can protect the premature infants with BPD. The target gene ADM of miRNA-574-3p may contribute to the prevention and treatment of BPD.

## Introduction

Although perinatal medicine is growing rapidly, the number of premature babies is still increasing [1]. For premature infants with impaired lung function, oxygen inhalation can save lives, but it may also increase the risk of getting bronchopulmonary dysplasia (BPD) [2].

Hyperoxia-induced reactive oxygen species (ROS) generation and lung inflammation leads to the development of BPD in premature infants [3]. BPD is a chronic lung disease (CLD) mainly found in premature infants with acute respiratory distress requiring mechanical ventilation (MV) and oxygen inhalation, it is still a major threat to premature infants [4].

MicroRNAs (miRs/miRNAs) are single-stranded, non-coding RNAs and evolutionarily conserved sequences of short chains (∼21–25 nucleotides). They act as endogenous repressors of gene expression by mRNA degradation and translation suppression [5].

They play an important role in cell differentiation, development, proliferation, signaling, inflammation and cell apoptosis [6]. miR-574-3p is one of miRs, adrenomedullin (ADM) is the target gene ADM of miR-574-3p. Previous studies had found that ADM-deficient fetal primary human pulmonary microvascular endothelial cells (HPMECs) increased oxidative stress, inflammation, and cytotoxicity compared with ADM-sufficient HPMEC upon exposure to hyperoxia [7]. ADM can reduce the production of ROS, indicating that ADM is an important regulator of oxidative damage [6].

However, it is not clear that ADM regulates the pathogenesis of BPD. We report here that the expression of ADM is up-regulated in premature infants with BPD. ADM has a protective effect on premature infants with BPD, and the present study has important clinical value.

## Materials and methods

### Micoarray expression profiling

Using Agilent Human 4x44K Gene Expression Microarrays v2 and miRCURY LNA™ microRNA Array to identify the differential expressed miRNA and its potential target genes (service provided by Kangchen Biotech, Shanghai, China). After hybridization and washing, the microarray slides were scanned with an Agilent DNA microarray scanner. The results of mRNA expression were extracted from Agilent Feature Extraction Software (version 11.0.0.1), then imported into the Agilent.

For further analysis, we used GeneSpring GX software (version 12.1). Using Axon GenePix 4000B to scan miRNA chip and GenePix Pro 6.0 software to read probes’ signal. Differently expressed genes were then identified through Fold Change (Fold Change ≥ 2) and *P*-value (*P*-value ≤0.05).

### Bioinformatics prediction

Using database miRBase and TargetScan to predict the potential target genes of miRNA. Only retaining these target genes which existed in both databases and had differential expression trends. Based on the expression value of miRNA and its target gene to calculate the Pearson’s correlation coefficient between them. The final pair of miRNA and target gene was identified through correlation coefficient ≤ −0.5 and *P*-value ≤0.05.

GO annotation and KEGG pathway enrichment [8] was done by R package cluster Profiler [9] and path view [10]. The threshold was Bonferroni adjusted *P*-value < 0.05.

### Subjects and sample collection

In the Department of Neonatology, Shanghai Children’s Hospital, we recruited 20 BPD premature infants and 20 age-matched non-BPD preterm infants according to the diagnosis standard of BPD [11], the premature infant (<32 weeks gestational age (GA)) had radiographic confirmation of persistant parenchymal lung disease and still needs the premise that auxiliary oxygen (invasive intermittent positive pressure ventilation (IPPV), nasal continuous positive airway pressure (N-CPAP)/non-invasive positive pressure ventilation (NIPPV), nasal cannula, Hood O_2_) at 36 weeks postmenstrual age (PMA) with one of the following FiO_2_ ranges/oxygen levels/O_2_ concentrations for ≥3 consecutive days to maintain arterial oxygen saturation in the 90–95% range.

Among the 40 premature infants, there were 26 males and 14 females, with GA (29.0 ± 3.64) weeks, 24 cases of birth weights ≤ 1500 g and 16 cases of 1500–2500 g. Their basic diseases were premature or accompanied by neonatal respiratory distress syndrome (NRDS). There were no perinatal infections among these infants, and the admission time was within 12 h after birth. In the BPD group of 20 premature infants, 14 were mechanically ventilated and 6 were inhaled with air-oxygen mixed through nasal catheter. Twenty non-BPD premature infants had no history of oxygen inhalation or no long-term history of oxygenation (air-oxygen mixed through nasal catheter inhalation, inhaled oxygen concentration < 30%, oxygen time < 3 days), and no BPD occurred among them.

The Shanghai Children’s Hospital Ethics Committee approved our research. After obtaining the informed consent of the guardian of the patients and signing a written document, we collected the peripheral blood of the premature infants as samples. Peripheral blood was collected from the peripheral blood of the 40 premature infants. The time points of peripheral blood collected from the premature infants in BPD group were 28 days after continuous oxygen use, and the time points of blood drawn from 20 age-matched normal premature infants were approximately 3 days after birth.

### Luciferase reporter gene analysis

The inhibitory effect of miRNA (hsa-miR-574-3p) on the target gene (ADM) was detected by double fluorescent reporter gene system. The site of interaction between miRNA and the target gene 3′UTR region could be further determined by site-directed mutagenesis. Experimental plasmid pMIR-REPORT Luciferase-ADM 3′UTR (WT) (H12118) and pMIR-REPORT Luciferase-ADM 3′UTR (MUT) (H12119) were built by Obio Technology (Shanghai) Corp., Ltd; pRL-CMV (H321, Promega); 293T cell line was derived from the cell bank of Chinese Academy of Sciences. The cell culture conditions were as follows: DMEM 10% FBS, 37°C, 5% CO_2_.

### Total RNA isolation and real-time q-PCR verification

According to the manufacturer’s instructions, we used TRIzol reagent (Invitrogen, Carlsbad, U.S.A.) to isolate total RNA from patients’ blood samples (BPD infants and non-BPD infants). RNA concentration can be quantified by means of a NanoDrop ND-2000 spectrophotometer (NanoDrop, Wilmington, DE). Next, reverse transcription (RT) reactions and real-time PCR were carried out in accordance with the methods described previously [12]. The expression coefficient of miRNA-574-3p and ADM relative to β-actin was calculated using the 2^−ΔΔ*C*_t_^ method. Table 1 lists the primers.

**Table 1:** Primers of ADM and β-Actin

Gene name	Primer	Sequence (5′–3′)
*ADM*	Forward	F: GTGGCCGAGGACTTTGATTG
	Reverse	R: CCTGTAACAACGCATCTCATATT
*β-Actin*	Forward	F: GGTTCGCTCGCCTTCCTA
	Reverse	R: CCCACTTATTCCACTTCTTTCG

### Statistics

Using Student’s *t* test to analyze experimental data. *t* test: *, *P*<0.05. **, *P*<0.01. The results of luciferase reporter gene were analyzed by two-way ANOVA. Each value appearing throughout the paper is the mean ± standard deviation that was tested by at least three independent experiments.

## Results

### Expression profile of BPD

**Figure 1:**
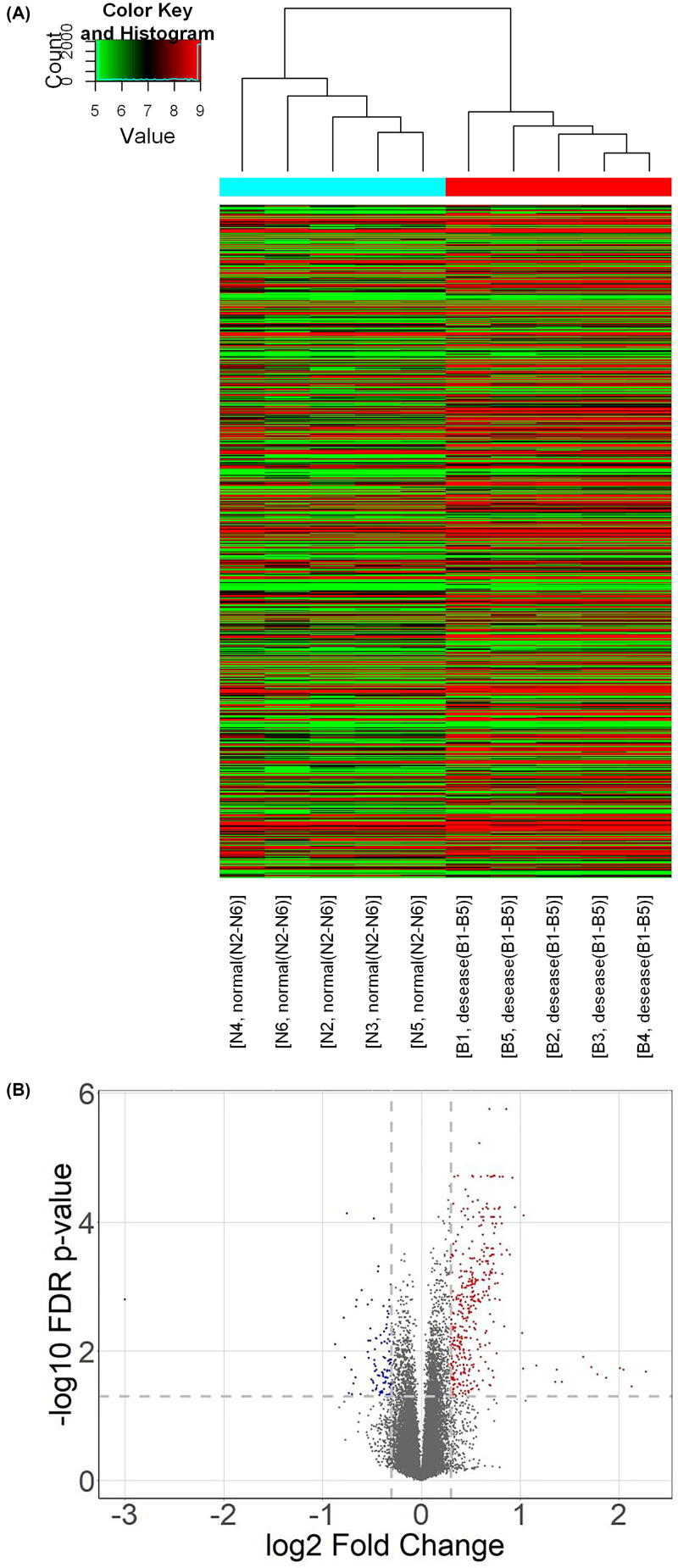
Heat map and the volcano plot of differentially expressing profile of BPD between BPD samples and non-BPD samples (**A**) Heat map of differentially expressing profile of BPD between BPD samples and non-BPD samples. In this plot, we show the expression level in each sample of all differential expressed probes. Red means up-regulated and green is down-regulated. After analyzing the two types of blood samples (BPD and non-BPD samples) by microarray expression, 516 probes were found to be differently expressed, of which 408 were up-regulated and 108 were down-regulated. The heat map represents the analysis results, wherein cut-off *P*-value <0.01 and fold change ≥ 2. The x-axis represents different samples and the y-axis represents genes. Among them, the highly expressed gene is represented by red spots, and the low expression gene is represented by green spots. Different samples are represented by different bar colors in the dendrogram; BPD samples are represented by red stripes and non-BPD samples are represented by green stripes. (**B**) The volcano plot of differentially expressing profile of BPD between BPD samples and non-BPD samples. In this plot, the red dots mean the significant up-regulated probes and blue dots are down-regulated probes. FDR *P*-value ≤0.05 and FC ≥ 2 were set as the threshold. Overall, we found 408 probes with up-regulated and 108 probes with down-regulated expression.

**Figure 2:**
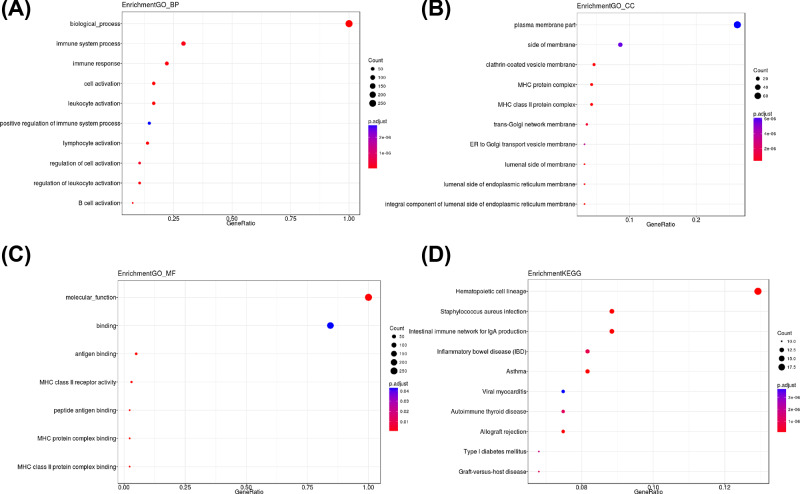
The dotplot of GO annotation and KEGG enrichment The y-axis is the GO or KEGG term. The gene ratio in x-axis is the relative abundance of genes in such term. (**A**) In GO annotation enrichment analysis, we found the differential expressed genes were enriched in immune system-related functions. (**B**) They were more likely to locate on membrane. (**C**) In the GO molecular function enrichment, we found differential expressed genes were enriched in binding function. (**D**) In KEEG pathway analysis, we found they were enriched in a lot BPD-related disease pathway such as asthma.

Using microarray expression to analysis data revealed that 516 probes expressed differently between the BPD samples and the non-BPD samples. Among these expressed differently probes were 408 probes with up-regulated expression and 108 probes with down-regulated expression (Figure 1, Supplementary Table S1). Meanwhile, for the level of miRNA, there were 37 up-regulated and 44 down-regulated (Figure 2, Supplementary Table S2).

### GO annotation and KEGG enrichment for differential expressed genes

The enrichment of GO in Biological Process showed that the expression of immune system, immune response and positive regulation of the immune system processes were significantly richer (Figure 2A). The results of Cellular Component showed that most of the differential expressed genes coding the membrane including trans-Golgi network membrane and lumenal side of endoplasmic reticulum membrane (Figure 2B). The Molecular Function results showed that most of proteins were the binding proteins, especially the MHC protein complex binding protein (Figure 2C).

**Figure 3:**
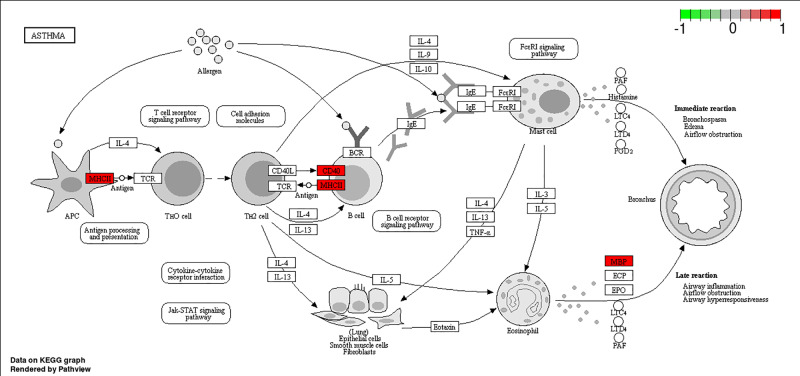
The asthma KEGG pathway This is an example for KEGG pathway enrichment analysis. In the plot, the red represents the up-regulated genes in our study including MHCII, CD40 and MBP.

The KEGG enrichment result showed that the asthma pathway was enriched (Figures 2D and 3).

### miR-574-3p and its target gene ADM

Based on the expression trends of miRNAs and their target genes, 73 pairs of miRNAs and mRNAs were selected from two miRNA target gene datasets as potential miRNA–mRNA pairs (Table 2, Supplementary Table S3). To make the result more accurate, the correlation coefficient of miRNA and mRNA was calculated based on their expression level. Sixty-five miRNA–mRNA pairs whose *P*-value ≤0.05 and correlation coefficient ≤ −0.5 were remained. According to relevant literature, ADM plays a key role in the development of BPD. ADM is an endogenous peptide that promotes angiogenesis while exerting antioxidant and anti-inflammatory effects. Previous study found that ADM-deficient HPMECs had significantly increased hyperoxia-induced ROS generation and cytotoxicity compared with AM-sufficient HPMECs. The hyperoxia damage in primary human fetal HPMECs lacking ADM increasing may be through activation of the Akt pathway [5]. Thus, for further research, we chose miRNA, miR-574-3p and its target gene ADM.

**Table 2:** The correlation coefficient of hsa-miR-574-3p and mRNA based on their expression level

miRNA	mRNA	Description	Correlation coefficient	*P*-value
hsa-miR-574-3p	NM_004633	*Homo sapiens* interleukin 1 receptor, type II (IL1R2), transcript variant 1, mRNA [NM_004633]	−0.7736	0.0087
hsa-miR-574-3p	NM_001124	*Homo sapiens* ADM, mRNA [NM_001124]	−0.7474	0.0130

### Luciferase reporter gene analysis

**Figure 4:**
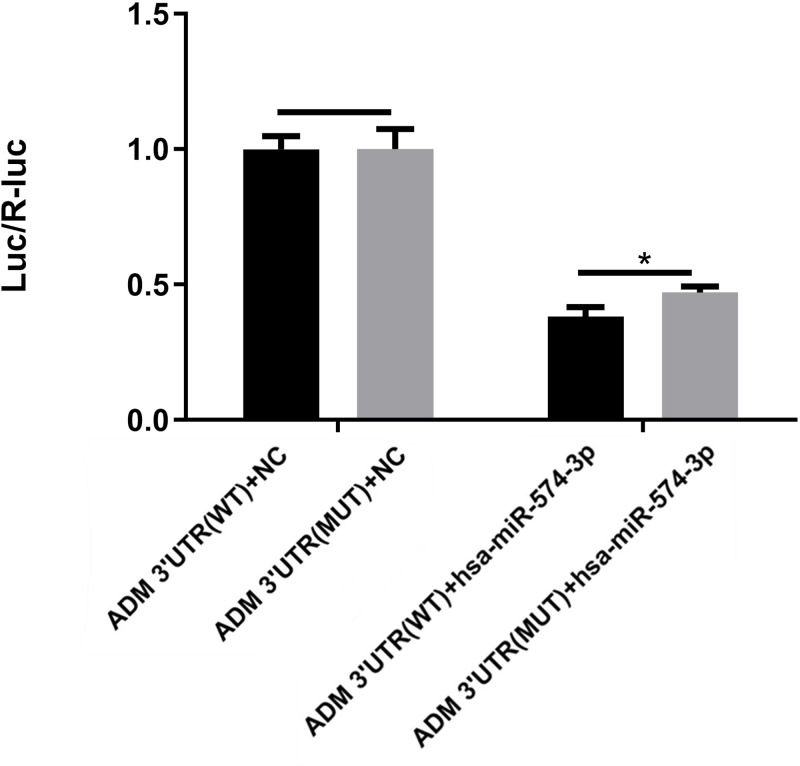
The relationship between miRNA (hsa-miR-574-3p) and its target gene ADM hsa-miR-574-3p can regulate the expression of luciferase with ADM 3′UTR (*P*<0.0001), and decrease it by 61.84%. But after the mutation of binding site, the regulatory relationship did not change much (*P*<0.0001), and only increased it by 8.90%. The results of two-way ANOVA analysis show that, there was no difference between ADM 3′UTR (WT) + NC group and ADM 3′UTR (MUT) + NC group. And ADM 3′ UTR (WT) + has-miR-574-3p group was significantly lower than ADM 3′UTR (MUT) + has-miR-574-3p group (*P*<0.05).

Luciferase reporter gene analysis showed that, hsa-miR-574-3p can regulate the expression of luciferase with ADM 3′UTR (*P*<0.0001) and decrease it by 61.84%. But after the mutation of binding site, the regulatory relationship did not change much (*P*<0.0001), and only increased it by 8.90%. The results of two-way ANOVA show that, there was no difference between ADM 3′UTR (WT) + NC group and ADM 3′UTR (MUT) + NC group. And ADM 3′ UTR (WT) + has-miR-574-3p group was significantly lower than ADM 3′UTR (MUT) + has-miR-574-3p group (*P*<0.05) (Figure 4).

### Real-time q-PCR was used to verify the expression of miR-574-3p and ADM in patients with BPD

**Figure 5:**
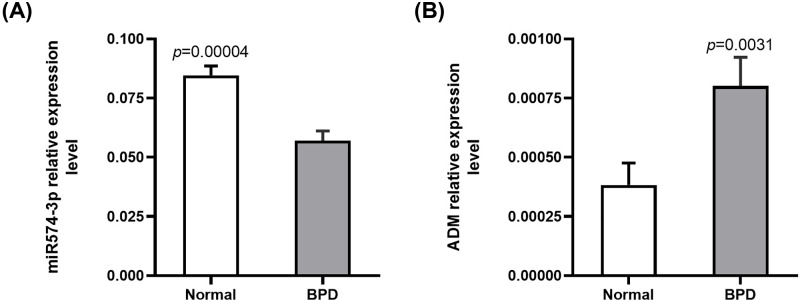
Real-time q-PCR verification of miRNA-574-3p and ADM expression in patients with BPD The expression of miR-574-3p in the blood of premature infants with BPD was significantly lower than that of premature infants without BPD (**A**), while the expression of ADM increased more significantly in the blood of premature infants with BPD (**B**). y-axis showed normalized relative expression level of miR-574-3p/ADM. *P*<0.05.

It was worth noting that the expression of miRNA-574-3p in the blood of premature infants with BPD was significantly lower than that of premature infants without BPD (Figure 5A), while the expression of ADM increased more significantly in the blood of premature infants with BPD (Figure 5B).

## Discussion

To date, we know very little about the regulatory function of miR-574-3p for ADM and its role in the development of BPD. Some scholars have found that ADM can play a role in infants with BPD. ADM is a vasoactive polypeptide that promotes lung development and has a protective effect. Peripheral blood of premature infants with BPD was detected by real-time q-PCR. The results showed that the expression of miR-574-3p was significantly decreased, while the expression of ADM was significantly increased.

Perinatal medicine has developed rapidly in recent decades, the improved neonatal care including widespread use of pulmonary surfactant (PS) and antenatal corticosteroids, and many advances in neonatal ventilation techniques, an increasing number of infants born at GAs less than 32 weeks survive [13]. The process of normal lung maturation includes canalicular phase and saccular/early phase, which are supposed to take place *in utero*, but preterm birth interrupts this process. The following three factors are likely to cause lung injury: first, perinatal infection after birth; second, inflammatory reaction; third, various oxygen therapy measures, such as inhaled oxygen, excessive oxygen concentration in MV, nasal cavity continuous positive airway pressure (NCPAP) and humidified high-flow nasal cannula (HHFNC) [14,15]. Hence, the incidence of BPD in premature infants has remained the same or even increased slightly [16].

Once discharged from NICU, infants with BPD are at a high risk of re-hospitalization due to higher susceptibility of viral infections, decreased nutritional state or poorer neurological outcome, leading to increased healthcare utilization and costs [17]. BPD is a disease secondary to premature birth destructing lung development [18]. In the United States, BPD ranks first in infants’ CLD and third in children’s CLD, but its pathogenesis is not fully understood.

In 1993, miRNAs were first discovered during the genetic screening of nematode model organisms (*Caenorhabditis elegans*) to identify post-embryonic developmental defects. MiRNAs are a class of small non-coding RNA genes whose final product is a 22-nt functional RNA molecule. They play an important role in the regulation of target genes by binding to complementary regions of messenger transcripts to repress their translation or regulate their degradation [19]. MiRNAs are strongly implicated in the pathogenesis of many common diseases, including BPD.

It has recently been reported that miRNAs are involved in the regulation of hyperoxia-induced lung cell damage and cell apoptosis [20].

Over past years of research, recombinant miRNAs are involved in gene silencing and play an important role in various biological processes [21]. Our study found that ADM was already found play a role in BPD patients. Real-time q-PCR was used to detect the expression of miR74-3p and ADM in peripheral blood of preterm infants with BPD. The results showed that the expression of the former was significantly decreased, while the expression of the latter was significantly increased. Luciferase reporter gene analysis showed that, hsa-miR-574-3p can regulate the expression of luciferase with ADM 3′UTR (*P*<0.0001), and decrease it by 61.84%. But after the mutation of binding site, the regulatory relationship did not change much (*P*<0.0001), and only increased it by 8.90%. The results of two-way ANOVA show that, there was no difference between ADM 3′UTR (WT) + NC group and ADM 3′UTR (MUT) + NC group. And ADM 3′ UTR (WT) + has-miR-574-3p group was significantly lower than ADM 3′UTR (MUT) + has-miR-574-3p group (*P*<0.05). It is speculated that hsa-miR-574-3p may also regulate the expression of luciferase through other binding sites, but not the only site. The above experimental results show that ADM plays a protective role in the development of BPD.

ADM is a vasoactive polypeptide that contains 52 amino acid residues and belongs to the calcitonin family, which includes calcitonin, amylinand, intermedin and calcitonin gene-related peptide (CGRP) [6]. ADM is a ubiquitous peptide that is expressed in all tissues of the body, including blood vessels and lungs [7]. Interestingly, studies have shown that ADM plays a crucial role in endothelial growth and survival [22,23]. ROS and inflammation play a key role in the pathogenesis of hyperoxia-induced lung disorders such as BPD and acute respiratory distress syndrome (ARDS) [24,25]. Zhang et al. reported that [6] ADM deficiency was associated with a significant increase in macrophage inflammatory protein-1 α (MIP-1α) and MIP-1β both in air and hyperoxic conditions. MIP-1a and MIP-1b are chemokines that can activate granulocytes and cause an inflammatory response. Several other investigators have suggested that MIP-1 chemokines may mediate acute [26] and chronic lung injury in mice under hyperoxic conditions [27,28].

Our research has some limitations. Ideally, we need to compare BPD samples with non-BPD samples using Agilent Human 4x44K Gene Expression Microarrays v2 and miRCURY LNA™ microRNA Array. After the above analysis, 516 probes were expressed differently. Among these probes, 408 of them were up-regulated and 108 down-regulated. However, at the miRNA level, 37 probes were up-regulated and 44 probes were down-regulated. ADM deficiency may potentiate hyperoxic injury in primary human fetal HPMEC via mechanisms entailing Akt activation [29]. Our results showed that the expression levels of miR-574-3p and ADM in the blood of premature infants with BPD were opposite. The former had increased expression and the latter had decreased expression. ADM may protect premature infants with BPD by antioxidation and anti-inflammation. However, it is unclear whether this is the primary effect of ADM on BPD, so this effect remains to be elucidated.

## Conclusion

In conclusion, the increased expression of ADM regulated by miR-574-3p can protect the premature infants with BPD and provide new ideas for the prevention and treatment of BPD.

## Supplementary Material

Supplementary Table S1-S3

## Data Availability

During the study, the data used and/or analyzed by the corresponding author were reasonable and compliant.

## Abbreviations

ADM: adrenomedullin
BPD: bronchopulmonary dysplasia
CLD: chronic lung disease
GA: gestational age
GO: gene ontology
HPMEC: human pulmonary microvascular endothelial cell
KEGG: kyoto encyclopedia of genes and genomes
MIP-1α: macrophage inflammatory protein-1 α
MIP-1β: macrophagein flammatory protein-1 β
miRNA/miR: microRNA
MV: mechanical ventilation
NICU: neonatal intensive care unit
q-PCR: quantitative PCR
ROS: reactive oxygen species
RT-PCR: real-time polymerase chain reaction
